# Long-term effects of cerebellar anodal transcranial direct current stimulation (tDCS) on the acquisition and extinction of conditioned eyeblink responses

**DOI:** 10.1038/s41598-020-80023-8

**Published:** 2020-12-31

**Authors:** Otilia Kimpel, Thomas Hulst, Giorgi Batsikadze, Thomas M. Ernst, Michael A. Nitsche, Dagmar Timmann, Marcus Gerwig

**Affiliations:** 1 Department of Neurology, Essen University Hospital, University of Duisburg-Essen, Hufelandstrasse 55, 45147 Essen, Germany; 2 Department of Endocrinology, University Hospital, University of Würzburg, Würzburg, Germany; 3 Erasmus University College, Rotterdam, The Netherlands; 4 Department of Psychology and Neurosciences, Leibniz Research Centre for Working Environment and Human Factors, Dortmund, Germany; 5 Department of Neurology, University Medical Hospital Bergmannsheil, Bochum, Germany

**Keywords:** Neuroscience, Physiology, Neurology

## Abstract

Cerebellar transcranial direct current stimulation (tDCS) has been reported to enhance the acquisition of conditioned eyeblink responses (CR), a form of associative motor learning. The aim of the present study was to determine possible long-term effects of cerebellar tDCS on the acquisition and extinction of CRs. Delay eyeblink conditioning was performed in 40 young and healthy human participants. On day 1, 100 paired CS (conditioned stimulus)–US (unconditioned stimulus) trials were applied. During the first 50 paired CS–US trials, 20 participants received anodal cerebellar tDCS, and 20 participants received sham stimulation. On days 2, 8 and 29, 50 paired CS–US trials were applied, followed by 30 CS-only extinction trials on day 29. CR acquisition was not significantly different between anodal and sham groups. During extinction, CR incidences were significantly reduced in the anodal group compared to sham, indicating reduced retention. In the anodal group, learning related increase of CR magnitude tended to be reduced, and timing of CRs tended to be delayed. The present data do not confirm previous findings of enhanced acquisition of CRs induced by anodal cerebellar tDCS. Rather, the present findings suggest a detrimental effect of anodal cerebellar tDCS on CR retention and possibly CR performance.

## Introduction

Transcranial direct current stimulation (tDCS) can alter cortical excitability and enhance neuronal plasticity^1,2^, an important physiological foundation of learning and memory formation. tDCS of primary motor cortex (M1) has been shown to improve motor learning^3,4^, and has become a promising option to enhance the beneficial effects of motor training in various neurological disorders^5–8^.

tDCS modulates learning-related plasticity not only in M1, but likely also in the cerebellar cortex^9,10^. Cerebellar tDCS was found to improve adaptive learning in arm reaching tasks in young and elderly healthy participants^11–13^. A more recent study in mice showed that the deletion of long-term potentiation (LTP) of Purkinje cells eliminates cerebellar tDCS effects on vestibulo-ocular reflex (VOR) habituation^14^, and thus delivers mechanistic information about the plasticity effects of cerebellar tDCS. Initial findings on the effects of cerebellar tDCS in cerebellar patients, however, are partly contradictory^15–18^. Cerebellar tDCS effects, moreover, show a significant degree of variability in healthy participants in reach adaptation tasks^19^.

Delay eyeblink conditioning is a motor learning task that critically depends on the integrity of the cerebellum^20–23^. In this task, an initially neutral conditioned stimulus (CS), commonly a tone, is repeatedly presented with an unconditioned stimulus (US), for example an air-puff directed to the eye. After repeated CS–US pairings participants learn that the CS predicts the occurrence of the US, and close their eye after onset of the CS and prior to occurrence of the US. We found that cerebellar tDCS of the cerebellum modulates delay eyeblink conditioning in healthy participants^24^. Anodal tDCS resulted in faster and enhanced acquisition of CRs, whereas cathodal stimulation impeded acquisition. The application of anodal tDCS during acquisition, however, led to significantly earlier CR onset, that is CRs were less appropriately timed. In a follow up study, we were unable to reproduce our initial findings^25^. Study designs, however, were not identical. In our first study, tDCS started at the beginning of the acquisition phase, whereas tDCS was started during an initial pseudo-conditioning phase and prior to the beginning of the acquisition phase in the second study. Furthermore, reinforcement rate was 100% in the first study, and 70% in the second study. Both aspects of the second study could have compromised tDCS effects. tDCS during pseudo-conditioning might have resulted in unforeseen learning during this stage, and interference in the acquisition stage, and the reduced reinforcement rate might have strengthened such an interference effect. The aim of the present study was to confirm our initial findings using the same experimental eyeblink conditioning set-up and paradigm, including the same reinforcement rate and onset of cerebellar tDCS. In the initial study a very long stimulation time of 42.9 min was used^24^. Because significant tDCS effects on CR acquisition were present within less than 10 min, decision was made to apply a more conventional stimulation time of 24.2 min. In addition to immediate effects, long-term effects of cerebellar tDCS on the acquisition and extinction of conditioned eyeblinks were also evaluated across multiple days.

## Results

### Unconditioned responses

Group mean values ± standard deviation (SD) of unconditioned response (UR) onset in unpaired trials in the initial pseudo-conditioning phase were 45.2 ± 9.9 ms in the anodal group and 51.3 ± 15.1 ms in the sham group (p = 0.14; unpaired t test). Group mean values ± SD of UR peak time were 103.8 ± 13.1 ms in the anodal group, and 107.8 ± 7.2 ms in the sham group (p = 0.61). Group mean UR duration was 119.3 ± 34.8 ms in the anodal group and 117.8 ± 49.9 ms in the sham group (p = 0.91). None of the trials had to be excluded, and analysis was based on 10 URs in each of the participants.

### CR incidence

**Figure 1:**
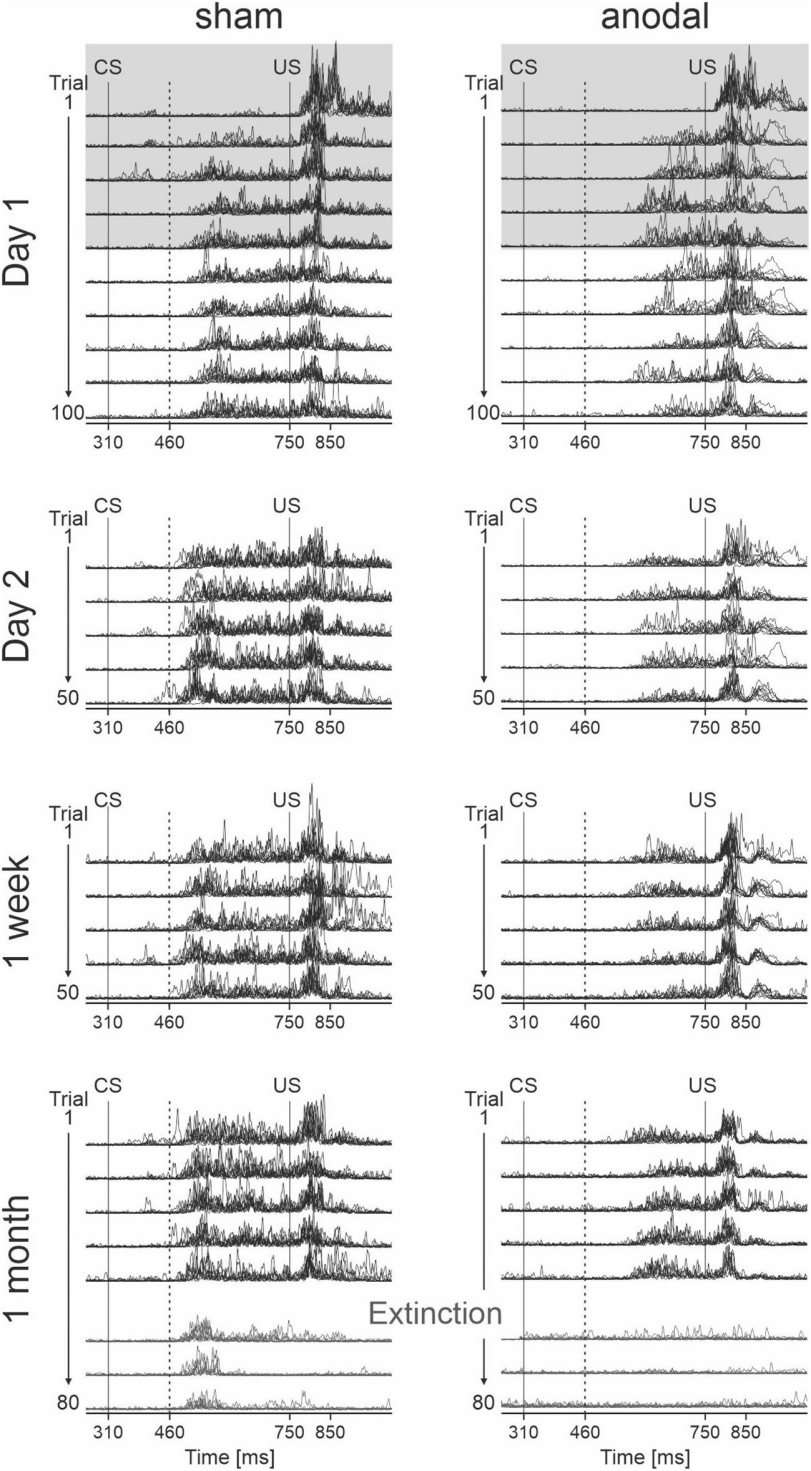
Individual examples of representative eyeblink recordings following sham and anodal tDCS across the four recording days. Rectified and filtered EMG-data of the orbicularis oculi muscle are shown. EMG recordings of 10 consecutive trials are superimposed. Day 1 is shown on the top, day 29 (1 month after stimulation) on the bottom. Duration of tDCS on day 1 is shaded in gray. Paired CS–US trials are shown in black, extinction trials are shown in green. The solid vertical lines indicate the onset of the CS and US, respectively. Responses occurring within the 150 ms interval after CS onset (dotted line) were considered alpha-responses. See “Methods” for further details.

The main findings are illustrated in Fig. 1 showing eyeblink recordings in two individual participants following sham and anodal stimulation across the four days. On day 1, the sham-stimulated participant and the verum-stimulated participant acquired conditioned responses (CRs) to a similar extent. In paired trials on subsequent days, CRs occurred later and were of smaller size in the verum-stimulated participant compared to the sham-stimulated participant. Furthermore, in the extinction phase one month after stimulation (day 29) the number of CRs was reduced in the verum-stimulated participant compared to the sham-stimulated participant.

**Figure 2:**
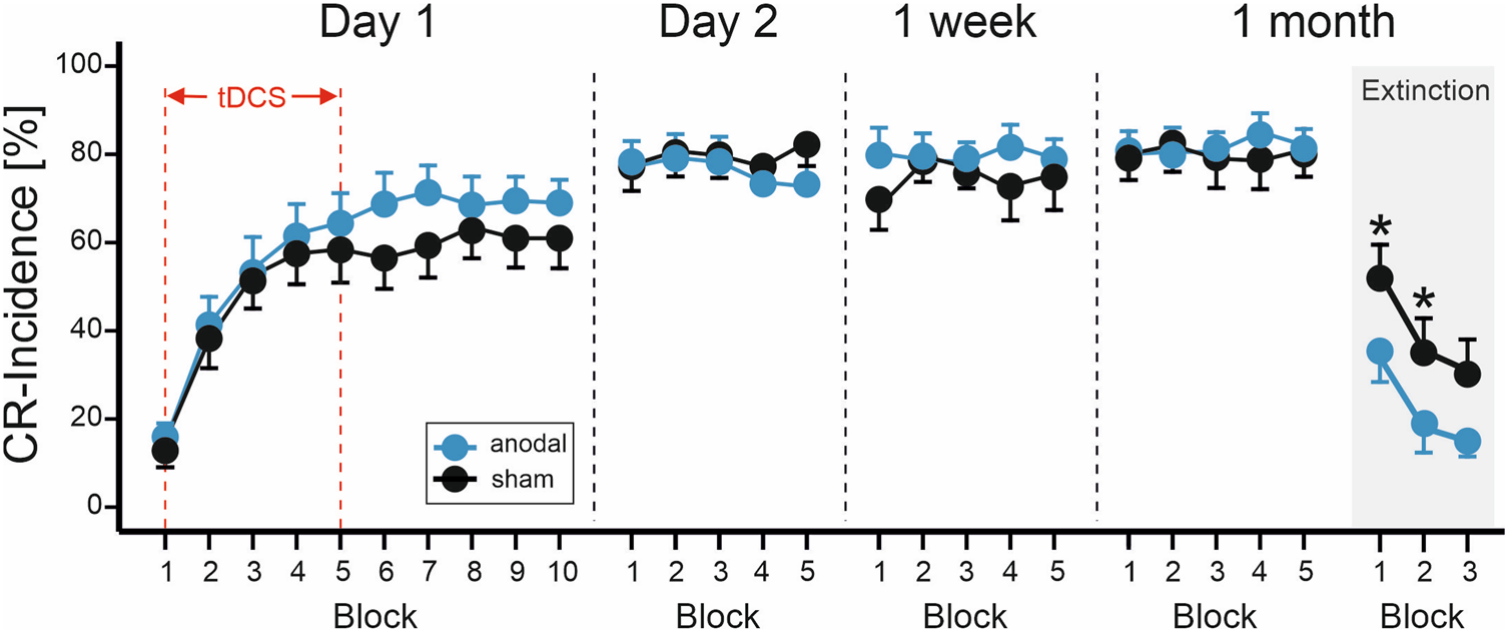
Group mean percentage CR incidence and standard errors (SE) on days 1, 2, 8 (1 week) and 29 (1 month). Ten blocks a 10 paired CS–US trials were presented on day 1, five blocks a 10 paired CS–US trials on days 2, 8 and 29. In addition, 3 blocks a 10 CS-only extinction trials were presented on day 29. Blue filled circles = anodal group, filled black circles = sham group. * indicates significant effects in post-hoc pairwise comparisons.

Group mean percentage of CR incidences ± standard errors (SE) across the four days in the two stimulation groups are shown in Fig. 2. On day 1, CR incidences increased in both stimulation groups across blocks. In the last five acquisition blocks, CR incidences were numerically higher in the anodal cerebellar stimulation group compared to the sham group. Mean total CR incidence on day 1 was 58.5% ± 5.0% in the anodal group compared to 52.2% ± 5.0% in the sham group. Mixed model ANOVA revealed a significant block effect [F(9,342) = 36.7; p < 0.0001]. The stimulation by block effect [F(9,342) = 0.55; p = 0.84] and the stimulation effect were not significant [F(1,38) = 0.67; p = 0.41].

CR incidences showed a further increase on day 2 compared to day 1, with no further increases during day 8 and day 29 and no differences between stimulation groups. Across days 2, 8 and 29 the mean total CR incidences was 79.5% ± 4.9% in the anodal group, and 78.5% ± 4.9% in the sham group. Mixed model ANOVA revealed no significant effects of day [F(2,76) = 1.0; p = 0.37] and stimulation [F(1,38) = 0.025; p = 0.87], and no significant stimulation by day interaction effect [F(2,76) = 1.06; p = 0.35]. That is both stimulation groups showed no further increase of CR incidences across the three days irrespective of the stimulation modality.

Regarding extinction, Fig. 2 shows a decrease of CR incidences across extinction blocks 1–3 on day 29 in both stimulation groups. Mean CR incidence in the extinction phase was lower in the anodal group (22.3% ± 5.3%) than in the sham group (39.8% ± 5.3%). Mixed model ANOVA revealed a significant block effect [F(2,76) = 17.6; p < 0.0001] and a significant stimulation effect [F(1,38) = 5.4; p = 0.026]. The stimulation by block interaction effect was not significant [F(2,76) = 0.083; p = 0.92]. Post-hoc pairwise comparisons, revealed a significant mean difference between the anodal and sham stimulation groups in the first extinction block (p = 0.032; mean difference of − 19.0% ± 8.6%) and the second extinction block (p = 0.047; mean difference of − 17.5% ± 8.6%). The mean difference of − 16.0% ± 8.6% in the third extinction block was not significant (p = 0.069).

### CR area

**Figure 3:**
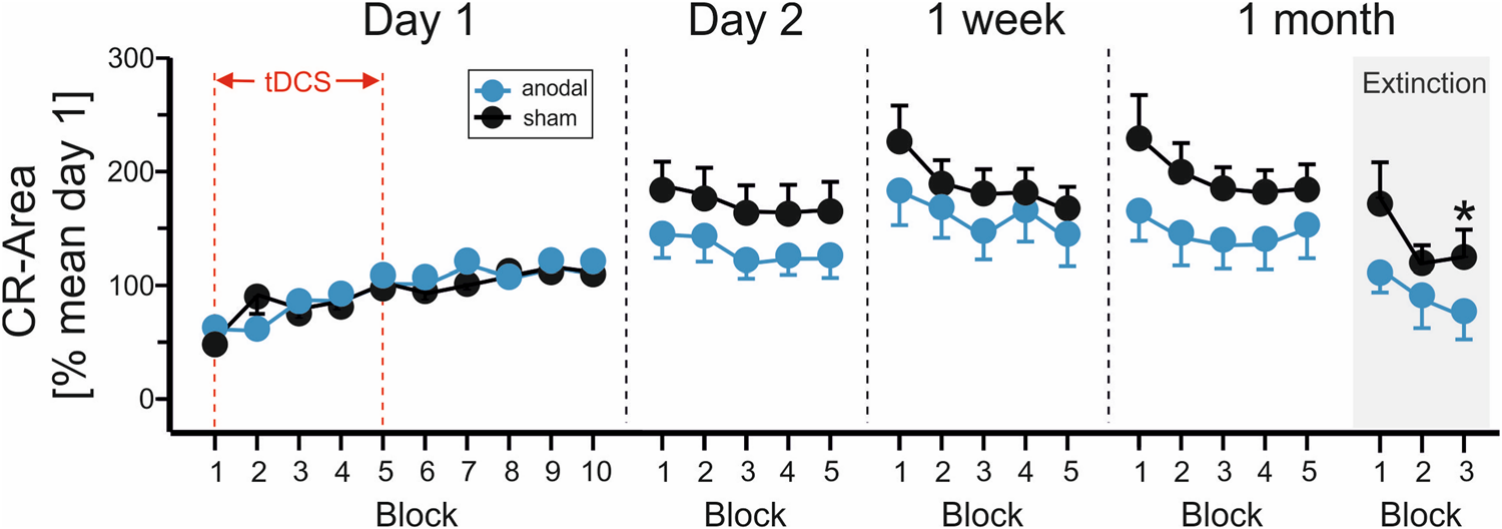
Group mean percentage CR area and standard errors (SE) on days 1, 2, 8 (1 week) and 29 (1 month). Ten blocks a 10 paired CS–US trials were presented on day 1, five blocks a 10 paired CS–US trials on days 2, 8 and 29. In addition, 3 blocks a 10 CS-only extinction trials were presented on day 29. Note that the number of participants per block varies because of lack of CRs in individual blocks. Blue filled circles = anodal group, filled black circles = sham group. * indicates significant effects in post-hoc pairwise comparisons.

On day 1, mean percentage CR area was not different between groups (anodal group: 95.5% ± 1.7%; sham group: 92.9% ± 14.7%) (Fig. 3). CR area increased across blocks in both groups. Mixed model ANOVA showed a significant block effect [F(9,303.25) = 6.6; p < 0.0001]. The stimulation by block interaction effect was not significant [F(9,303.25) = 0.86; p = 0.56]. The stimulation effect (that is anodal vs. sham stimulation) was not significant [F(1,31.7) = 1.1; p = 0.29].

Mean percentage CR area on days 2, 8 and 29 was smaller in the anodal group (145.2% ± 14.8%) compared to the sham group (187.3% ± 14.8%). CR area increased across days in both groups. The stimulation effect was close to significance [F(1,38) = 4.0; p = 0.052]. There was no significant effect of day [F(2,76) = 1.4; p = 0.25] and no significant stimulation by day interaction [F(2,76) = 0.31; p = 0.84].

In extinction trials on day 29, CR area was smaller in the anodal compared with the sham group (91.9% ± 21.5% vs. 156.6% ± 20.7%). CR area declined across extinction blocks in both groups. Mixed model ANOVA revealed a significant block effect [F(2,50.047) = 3.9; p = 0.027] and a significant stimulation effect [F(1,35.43) = 4.7; p = 0.037]. The stimulation by block effect was not significant [F(2,50.047) = 0.23; p = 0.79]. Post-hoc pairwise comparisons comparing the anodal and sham stimulation groups in each of the extinction blocks revealed a significant mean difference in the third block (p = 0.033; mean difference of − 74.1% ± 33.9%). The mean differences in the first extinction block (p = 0.053; − 62.8% ± 31.5%) and the second block (p = 0.093; − 57.2% ± 33.4%) were not significant.

### CR timing

**Figure 4:**
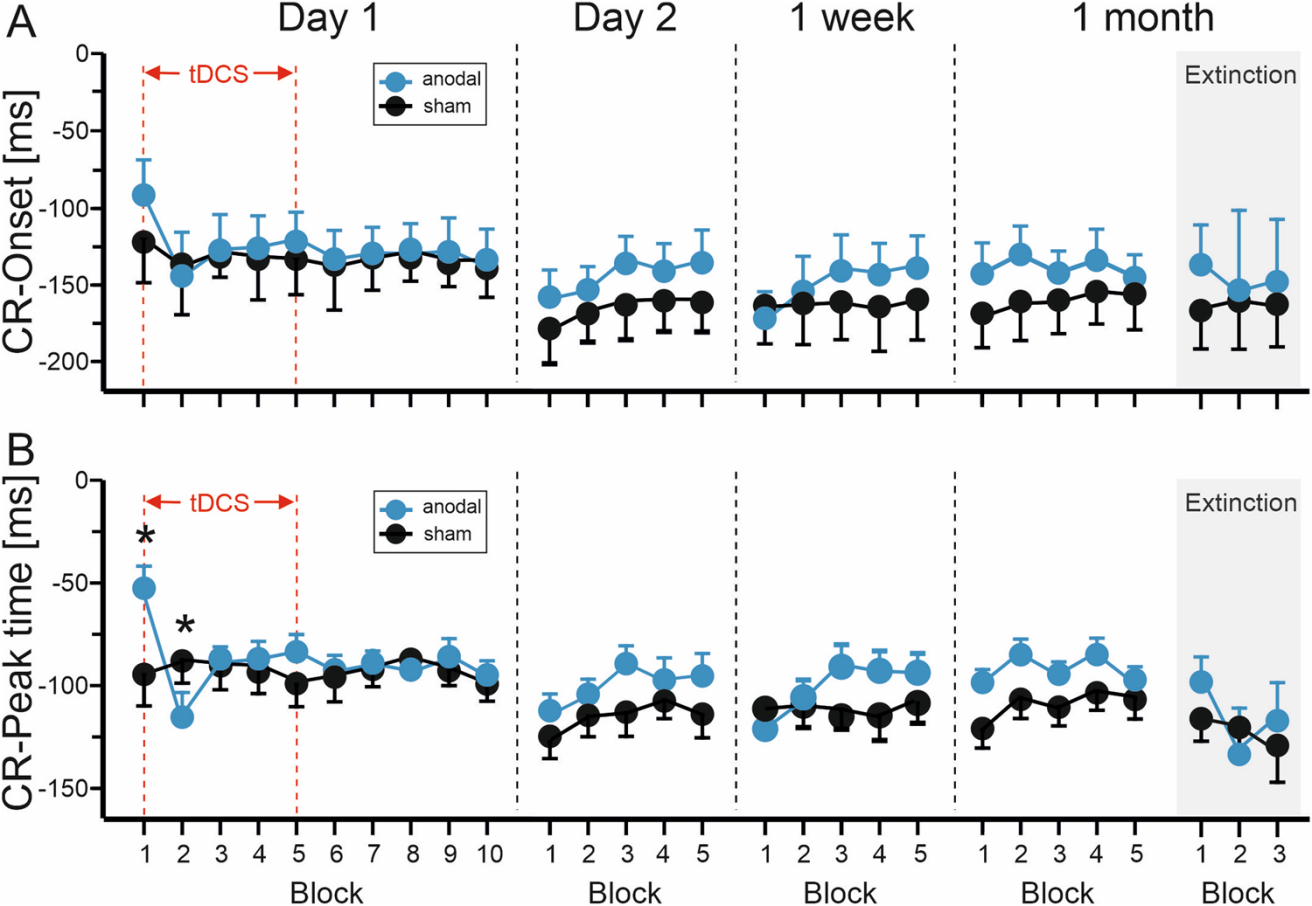
Group mean and standard errors (SE) of CR onset (**A**) and peak time (**B**) latencies on days 1, 2, 8 (1 week) and 29 (1 month). Ten blocks a 10 paired CS–US trials were presented on day 1, five blocks a 10 paired CS–US trials on days 2, 8 and 29. In addition, 3 blocks a 10 CS-only extinction trials were presented on day 29. Negative values refer to the time prior to the onset of the US (air-puff) set as 0 ms. Note that the number of participants per block varies because of lack of CRs in individual blocks. Blue filled circles = anodal group, filled black circles = sham group. * indicates significant effects in post-hoc pairwise comparisons.

Mean values of CR onset and peak time latencies across paired trials and extinction trials are shown in Fig. 4. On day 1, mean CR onset latencies were − 123.7 ± 7.5 ms (that is, prior onset of the US set as 0) in the anodal group and − 132.2 ± 7.5 ms in the sham group. In the very first block, CR onsets differed between the sham and anodal group, with CR onset being closer to US onset in the anodal group, therefore occurring later after CS onset compared to the sham group. CR onsets did not differ in subsequent blocks. Linear mixed model analysis revealed a significant block effect [F(9,300.2) = 2.8; p = 0.004]. The stimulation by block [F(9,300.2) = 1.0; p = 0.41] and the stimulation effects [F(1,38.6) = 0.64; p = 0.43] were not significant.

Considering days 2, 8 and 29, mean CR onset latencies were − 142.0 ± 7.3 ms in the anodal group and − 161.0 ± 7.3 ms in the sham group. CR onset latencies occurred numerically later in the anodal compared to the sham group. Analysis of CR onset latencies did not reveal a significant effect of day [F(2,76) = 1.0; p = 0.36]. The stimulation by day effect [F(2,76) = 0.50; p = 0.61] and the stimulation effect [F(1,38) = 3.2; p = 0.079] were not significant.

In extinction trials mean CR onset latencies were − 147.1 ± 10.1 ms in the anodal group and − 159.8 ± 9.6 ms in the sham group. Analysis of CR onset latencies did not reveal significant block [F(2,60.83) = 0.2; p = 0.81], stimulation by block [F(2,60.83) = 0.6; p = 0.55] or stimulation effects [F(1,36.39) = 0.83; p = 0.37].

CR peak time latencies showed similar results. On day 1, mean peak time latencies were − 88.7 ± 6.7 ms in the anodal group and − 93.3 ± 6.7 ms in the sham group. In the very first block, CR peak time occurred later after CS onset in the anodal compared to the sham group. In the second block CR peak time, however, occurred earlier in the anodal group. The block effect [F(9,301.04) = 0.99; p = 0.44], and the stimulation effect were not significant [F(1,39.02) = 0.24; p = 0.63]. The stimulation by block effect was significant [F(9,301.04) = 2.7; p = 0.005]. Pairwise comparisons between anodal and sham stimulation revealed a significant mean difference of 48.3 ± 16.7 ms in the first block (p = 0.004), and a significant mean difference of − 33.1 ± 13.8 ms in the second block (p = 0.018). Mean differences comparing blocks 3–10 were not significant (all p values > 0.2).

Considering days 2, 8 and 29, mean CR peak time latencies were − 98.4 ± 6.7 ms in the anodal group and − 112.9 ± 6.7 ms in the sham group. Mixed model analysis did not reveal significant effects of day [F(2,76) = 1.5; p = 0.23], stimulation by day [F(2,76) = 0.33; p = 0.72] or stimulation [F(1,38) = 2.3; p = 0.13].

In extinction trials mean CR peak time latencies were − 115.5 ± 10.3 ms in the anodal group and − 121.1 ± 9.7 ms in the sham group. Mixed model analysis did not reveal significant block [F(2,61.25) = 1.1; p = 0.35], stimulation by block [F(2,61.25) = 0.7; p = 0.50] or stimulation effects [F(1,37.97) = 0.16; p = 0.69].

### Alpha responses

The mean alpha-response count was 1.1 ± 1.5 in the anodal group and 1.2 ± 1.5 in the sham group. The group difference was not significant (p = 0.67; unpaired t test).

### Spontaneous blink rate

Spontaneous blinks were assessed within one minute both at the beginning and at the end of each day (Table 1). Comparison of spontaneous blink rates did not reveal significant differences between stimulation groups on each day (all p values > 0.5; unpaired t tests).

**Table 1:** Spontaneous blink rates (blinks per minute ± SD) as assessed at the beginning and at the end of each day in the anodal and sham group.

Blinks/min	Anodal	Sham
Day 1		
Beginning End	17.0 ± 4.0 17.0 ± 5.2	17.8 ± 4.1 17.9 ± 4.5
Day 2		
Beginning End	17.0 ± 3.7 16.4 ± 3.2	17.8 ± 4.1 17.9 ± 4.2
Day 8		
Beginning End	17.7 ± 4.1 18.1 ± 4.9	18.2 ± 5.1 18.0 ± 4.9
Day 29		
Beginning End	17.2 ± 3.5 17.1 ± 3.0	17.7 ± 3.8 17.5 ± 3.3

## Discussion

The present data confirm that anodal cerebellar tDCS has a modulatory effect on eyeblink conditioning. Different to previous results, however, we did not observe significant beneficial effects on acquisition learning. Rather, long-term detrimental effects of cerebellar tDCS were most prominent. Anodal tDCS impeded the long-term retention of these learned motor responses. Furthermore, timing and magnitude of conditioned responses seemed to be impaired.

We were unable to confirm our initial findings of significantly enhanced CR acquisition as a consequence of cerebellar anodal stimulation^24^. Similar to Beyer et al.^25^ CR incidences tended to be higher in the anodal group compared to sham stimulation, but this difference did not reach significance. The present and the two previous studies were performed using the same eyeblink conditioning set-up. The delay conditioning paradigm differed between the Beyer et al.^25^ and the Zuchowski et al.^24^ studies, but were the same in the present and the Zuchowski et al.^24^ studies. Thus, differences in findings cannot be explained by differences in tDCS onset or reinforcement rate. However, Zuchowski et al.^24^ stimulated throughout the acquisition phase of 100 paired CS–US trials, whereas in the present study tDCS stimulation stopped after the first 50 paired CS–US trials sticking to a more conventional time of stimulation of about 20 minutes^26^. This is unlikely to account for the lack of cerebellar tDCS effects during the first 50 acquisition trials, but may have had an impact on the second half of acquisition trials and long-term effects across the following weeks. In the present study, CR incidence was numerically higher in the anodal compared to the sham group in the last 50 paired trials. Numerically, however, there was no difference between the two groups in the first 50 acquisition trials. Of note, cerebellar tDCS effects were most prominent as early as the first 15 acquisition trials in our previous study (see Fig. 2B in Zuchowski et al.^24^). In that study, cathodal tDCS had also been applied and showed reduced conditioning rates. Polarity dependent effects of cerebellar tDCS on CR acquisition, however, were not confirmed in our follow-up study^25^. Zuchowski et al.^24^ used a one day paradigm and long-term effects of cerebellar tDCS were not assessed. In the present study, neither immediate nor long-term effects on the acquisition of conditioned eyeblink responses were observed.

The present findings on conditioned eyeblink acquisition are in good accordance with previous findings of cerebellar tDCS effects on reach adaptation, another motor learning task which is cerebellar dependent. Here initial findings of Galea et al.^11^ were not reproduced in later studies^18,27^. Again, one may argue that these studies used different reach adaptation set-ups and paradigms. Lack of reproducibility, however, was shown also in a later study by Galea and colleagues themselves in a carefully performed study using the same set-up and paradigm^19^.

In the present study anodal tDCS tended to impede performance parameters of conditioned eyeblink responses. Anodal tDCS of the cerebellum applied during initial acquisition seemed to reduce the increase in size of CRs in repeated conditioning sessions across four weeks, indicating a long-term detrimental effect. An augmentation of CR amplitudes during eyeblink conditioning is well known, and has already been reported in early studies investigating healthy human participants^28,29^. Accordingly, in the sham group, we found that the increase of CR incidences was accompanied by an increase in CR area. Tran et al.^30^ also found an increased CR area in healthy children parallel to an increased rate of conditioned responses, which was not observed in preterm children. In preterm children cerebellar development is impeded^31,32^ and reduced size of CRs was related to disordered cerebellar function. Furthermore, animal studies show that amplitudes of conditioned responses are diminished following cerebellar lesions^33^. It has been argued that the cerebellum is primarily engaged in the performance of conditioned responses^34–36^. Anodal tDCS is thought to increase the excitability of the cerebellar cortex^37,38^, and may lead to an increased inhibition of the cerebellar nuclei and therefore decreased cerebellar amplitudes.

Timing of CRs appeared also to be impeded by anodal tDCS. The difference was most prominent in the first 10 paired acquisition trials of day 1. To a lesser degree, this difference was also found on subsequent days, with CRs tending to occur later in the group which received anodal stimulation on the first day. In accordance with the present findings, Mitroi et al.^39^ reported significantly longer CR peak and onset latencies following anodal cerebellar stimulation compared to sham. This is in contrast to findings of our previous study that found that CRs occurred significantly earlier during anodal tDCS and throughout the acquisition phase^24^. Beyer et al.^25^, on the other hand, did not find any significant effects of cerebellar anodal tDCS on CR timing parameters. Thus, anodal cerebellar tDCS effects led to opposing effects on timing of conditioned responses.

Animal studies show that conditioned responses in delay eyeblink conditioning are dependent on pauses of Purkinje cell activity which results in less inhibition of the neural activity of the cerebellar nuclei just before the US onset and induces the generation of a well-timed CR^40,41^. In animal models large cortical lesions that involve the anterior lobe caused short-latency responses^42,43^ which were also reported in transgenic mice with impaired long-term depression (LTD) at the parallel fiber-Purkinje cell synapse^44^. Although less shifted forward, also in patients with cortical lesions of superior parts of the cerebellar hemisphere, mean CR onset occurred too early, on average 20 ms earlier than in controls^45^. tDCS effects are critically dependent on orientation of the dendrites and other neuronal structures to the electric field^46–48^ and the highly convoluted cerebellar cortex may explain opposing effects in different participant populations: inhibition of the cerebellar nuclei may be either ramped up or down (or not changed at all) depending on the total net effects of cerebellar tDCS stimulation, and may therefore result on opposing effects on CR timing (or none at all). Furthermore, cerebellar tDCS may affect the cell populations in the different cerebellar layers to various extents which can result in a heterogeneous compound effect^38^.

Most importantly, anodal cerebellar tDCS during early acquisition let to impeded retention of conditioned eyeblink responses. CR incidences were significantly less in the first and second block of the extinction phase on day 29 in the anodal stimulated group compared to sham. Decrease of CR incidences across extinction trials, however, was not different between groups, that is extinction learning did not appear to be affected by anodal tDCS during acquisition learning. In the Zuchowski et al.^24^ study no detrimental effects on retention in the extinction phase was observed. Extinction, however, was tested within the same session as acquisition, and there was no time for consolidation. Similar to the present study, no effects on extinction learning were found. Likewise, we did not observe that anodal cerebellar tDCS applied during the extinction phase led to changes of extinction learning^25,49^. This, however, does not exclude that the cerebellum contributes to extinction. There is good evidence in the rodent literature that the cerebellum contributes to extinction^20,50,51^. Consistent with the present findings, Jongkees et al.^52^ reported not only performance impairment during anodal cerebellar tDCS, but also long-term effects of stimulation in a serial reaction time task. The authors investigated effects of cerebellar tDCS on motor response selection and sequence acquisition, and found an increase in overall reaction time during stimulation. This group difference was not only present as an immediate effect during tDCS, but reappeared at 24-h follow-up when participants performed the task without stimulation. Results point to a detrimental effect of anodal cerebellar tDCS on sequence consolidation and retention. For motor sequence learning, the primary motor cortex is known to be relevant in the acquisition stage, as excitability enhancement of this area with anodal tDCS improves learning^3,4^. Since motor cortex excitability can be reduced by anodal cerebellar tDCS, this might explain performance-reducing effects for this task. Regarding eyeblink conditioning, retention is thought to take place at least in part within the cerebellar nuclei, with initial learning taking place primarily in the cerebellar cortex^53–55^. The excitability alteration of the cerebellar cortex evoked by anodal stimulation may impede the transfer of learning from the cerebellar cortex to the cerebellar nuclei. Learning related plasticity is likely not confined to the cerebellum. Thus, changes in activity in cerebello-cerebral networks involved in eyeblink conditoning may also play a role^56–61^.

The present data confirm that cerebellar anodal tDCS modulates cerebellar-dependent motor performance and motor learning processes. Findings, however, provide further evidence that cerebellar tDCS effects lack robustness and are difficult to predict^19,25^. As outlined above, likely the most important factor is the highly convoluted cerebellar cortex which, because of the direction dependency of tDCS effects, likely results in opposing tDCS effects across stimulated cerebellar lobules in an individual participant, which makes the net effect hard to predict^62^. Interindividual differences due to anatomical variability have been shown to influence the direction of the electric field and current flow in relation to the orientation of the neuronal tissue^63–65^. The involvement of different cerebellar cell populations as well as the anatomical complexity of the cerebellar cortex may also play a role, and may explain why different tDCS protocols result in different behavioural outcomes^38^. Furthermore, heterogeneous tDCS effects have been related to the BDNF polymorphism (brain-derived neutrotropic factor), a factor relevant for synaptic plasticity^66–68^. A specific role of the BDNF polymorphism in eyeblink conditioning has been related to specific firing patterns of Purkinje cells^69^. tDCS effects have also been reported to depend on individual sensitivity to non-invasive brain stimulation^70^. Labruna et al.^70^ found that sensitivity of M1 to transcranial magnetic stimulation (TMS) pulses correlated with tDCS effects on M1 on visuomotor adaptation. It will be of interest for future studies to investigate whether the sensitivity to effects on cerebellar-brain-inhibition (CBI) correlates with cerebellar tDCS effects on motor learning. Finally, differential tDCS effects on distinct cell populations in the cerebellar cortex have to be taken into account, which may lead to different tDCS effects depending on stimulation parameters^38^.

The present study has some limitations. The study may be underpowered. Although 20 participants per stimulation group were included, stimulation effects in the acquisition phase may only occur in larger study populations. Furthermore, reduced retention effects were present 4 weeks after stimulation. It would be of interest to show that similar effects can be observed on the day following tDCS, that is after one night of consolidation. Moreover, tDCS was restricted to anodal stimulation compared to sham, without a cathodal stimulated group, and the time of stimulation was shorter compared to our initial study. Finally, possible individual factors like anatomical variability at the site of stimulation have not been considered.

## Conclusions

Findings of the present study suggest a detrimental effect of anodal cerebellar tDCS on the performance and timing of learned motor responses. In addition, retention was reduced as assessed four weeks after stimulation, indicating long-term detrimental effects. Enhanced acquisition of conditioned motor responses by anodal tDCS as previously reported was not confirmed. Future studies are needed to understand the factors predicting the outcome of cerebellar tDCS effects on motor performance and learning in individual participants.

## Methods

### Participants

A total of 40 young, healthy and right handed participants took part in the study. They were randomly assigned to two stimulation groups. One group (10 males, 10 females, mean age 21.7 ± SD 2.5 years received anodal tDCS, the second group (10 males, 10 females, mean age 22.8 ± SD 3.5 years) received sham tDCS. None of the participants had a history of neurological disease. None were taking centrally acting medication. Neurological examination, including ataxia rating scales^71,72^, was performed on the first day of the experiment, and were unremarkable. All participants were naive to eyeblink conditioning and tDCS. In each participant, hearing thresholds (dB SPL) were determined using a tone of 1 kHz. Values corresponded to normal age limits in all participants. The study was approved by the ethics committee of the Essen University Hospital and all methods and experiments were performed in accordance with relevant guidelines and regulations. Oral and written informed consent was obtained from all participants.

### Experimental procedure

**Figure 5:**
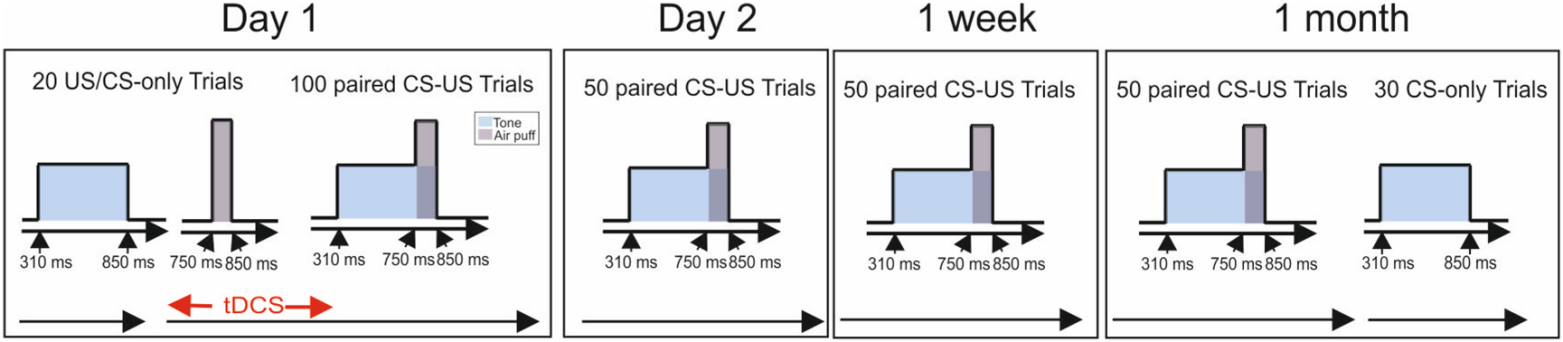
Experimental protocol. At the beginning of day 1, 10 CS-only trials and 10 US-only trials were presented in a pseudorandom sequence (pseudo-conditioning), followed by 100 paired CS–US trials. tDCS was started after the pseudo-conditioning phase and lasted throughout the first 50 paired CS–US trials. On days 2, 8 (1 week) and 29 (1 month), 50 paired CS–US trials were given. On day 29 (1 month) the 50 paired CS–US trials were followed by 30 CS-only extinction trials.

Delay eyeblink conditioning was performed on four days: day 1, day 2, after one week (day 8), and after one month (day 29) (Fig. 5). On day 1, following a pseudoconditioning phase of 10 CS-only and 10 US-only trials presented in pseudorandom order, 100 paired CS–US trials were applied. During the first 50 paired CS–US trials, 20 participants received anodal cerebellar tDCS, and 20 participants received sham stimulation. On days 2, 8 and 29, 50 paired CS–US trials were applied. This was followed by 30 CS-only extinction trials on day 29.

### Eyeblink conditioning

Participants sat comfortably in a chair with both arms resting on armrests. During eyeblink conditioning a silent movie was shown on a screen positioned in front of the participants to maintain vigilance. Conditioned responses (CRs) were recorded from orbicularis oculi muscles bilaterally via surface electrodes which were fixed to the lower eyelid and to the nasion. Signals were fed to EMG amplifiers (sampling rate 1000 Hz, band pass filter frequency between 100 Hz and 2 kHz), full wave-rectified and further low pass-filtered offline (100 Hz). A standard delay eyeblink conditioning protocol was used according to Gormezano and Kehoe^73^. The CS, a neutral tone (1 kHz, 70 dB SPL, duration 540 ms), was provided via headphones and superimposed on a continuous white noise (60 dB SPL) to mask environmental noise. An air-puff (duration 100 ms, intensity 400 kPa at source, 110 kPa at nozzle) was used as US. The US was directed laterally to the outer canthus of the right eye through a nozzle mounted on a helmet worn by the participants. The CS started 310 ms after onset of each trial, preceded the US onset by a fixed time interval of 440 ms and coterminated with the US. The intertrial interval varied randomly between 20 and 35 s.

Conditioned eyeblink responses were semiautomatically analyzed using a custom made software^74^. CRs were identified within the CS–US window. Responses occurring within the 150 ms interval after CS onset were considered as reflexive responses to the tone (alpha-responses) and not rated as CRs^75^. Trials with spontaneous blinks occurring prior CS onset were excluded from the analysis^76^. Rectified EMG recordings were filtered using a series of non-linear Gaussian filters. CRs were identified when EMG activity reached 7.5% of the EMG maximum in each recording with a minimum duration of 20 ms and a minimum integral of 1 mV*ms. All trials were visually inspected and implausible identification of CRs was manually corrected. The total number of paired (and unpaired extinction) trials was subdivided into blocks of 10 trials each. The number of CRs was expressed as the percentage of trials containing responses with respect to each block of 10 trials (percentage CR incidence) and the total number of trials (total percentage CR incidence). In addition, CR onset, peak time and area were analyzed. CR onset and peak time were expressed as negative values prior US onset set as 0 ms. CR peak time was defined at the time of maximum amplitude before US onset in paired trials. Mean baseline area was assessed in an interval of 100 ms prior US onset in each trial and subtracted from CR area. CR area was expressed as percentage of mean CR area across all CRs on day 1 in each participant, set as 100%. CR area was normalized in order to allow comparisons of changes across time. The frequency of spontaneous blinks was measured on each day within 1 min at the beginning and the end of the experiment. The number of alpha-blinks was assessed.

### Cerebellar transcranial direct current stimulation

Cerebellar tDCS was applied using a neuroConn DC Stimulator Plus (serial number 0371; neuroConn GmbH). Two conductive carbon–rubber electrodes (5 cm × 7 cm, surface area 35 cm^2^) and conductive electrode paste (Weaver ten20) were used. The cerebellar electrode was centered 3 cm lateral to the inion in a vertical position over the right cerebellar hemisphere^24^. The return electrode was placed on the ipsilateral buccinator muscle^37^. The current of anodal tDCS was set to 2 mA^77^ with a ramp-like fade-in and fade-out stimulation of 30 s (current density 0.057 mA/cm^2^). Stimulation started with the acquisition phase on day one and was performed throughout 50 of the 100 paired CS–US trials (Fig. 5). The overall duration of stimulation was 24 min and 12 s including the fade-in and fade-out time. In the sham condition the same fade-in of 30 s was used followed by 48.4 s. of tDCS and a fade-out time of 30 s. The modality of stimulation was unknown to the participants as well as to the investigator. Cerebellar tDCS was well tolerated. Some participants reported a mild tingling at the beginning of the stimulation. tDCS protocols were identical to Zuchowski et al.^24^ with two exceptions: (1) stimulation time was reduced from 42.9 min in the previous study to a more conventional stimulation time of about 20 min in the present study, (2) conductive electrode paste was used instead of sponge electrodes soaked in saline solution.

### Data analysis

Statistical analyses were performed in SPSS (SPSS Statistics 25.0; IBM). First, timing parameters of unconditioned eyeblink responses were analyzed using unpaired t tests. Next, linear mixed model analyses were performed. To assess immediate tDCS effects on CR acquisition learning on day 1, CR incidence was used as dependent variable, block (1–10; 10 blocks of 10 paired trials) as within subject factor and stimulation group (anodal vs. sham) as between subject factor. To assess long-term tDCS effects on CR incidence across days, CR incidence was used as dependent variable, day (day 2, day 8, day 29) as within subject factor and stimulation group (anodal vs. sham) as between subject factor. To analyze tDCS effects on extinction, CR incidence was used as dependent variable, extinction block (1–3; 10 blocks of 10 extinction CS-only trials) as within subject factor and stimulation group (anodal vs. sham) as between subject factor. Similar mixed model analyses were performed considering CR onset, peak time and area as dependent variable. Level of significance was set at p < 0.05.
